# “One counselling strategy does not fit all”: Midwives’ experiences of providing contraceptive counselling to girls and young women with ADHD - a Swedish qualitative interview study

**DOI:** 10.1186/s12905-026-05043-4

**Published:** 2026-10-26

**Authors:** Margareta Johansson, Alida Haglund, Nina Asplin

**Affiliations:** 1 Department of Women’s and Children’s Health, Uppsala University, Uppsala, Sweden; 2 Department of Health Sciences, Lund University, Lund, Sweden; 3 Department of Obstetrics and Gynecology, Danderyds Hospital, Stockholm, Sweden; 4 Department of Women’s Health Care, Akademiska University Hospital, Uppsala, Sweden

**Keywords:** ADHD, Contraceptive counselling, Interview, Midwives, Youth clinic, Women

## Abstract

**Background:**

Despite accessible services, unmet contraceptive needs persist among young women in Sweden. Young women with ADHD may have diverse sexual and reproductive health needs, and previous research has identified challenges related to healthcare access, communication, and contraceptive use. However, less is known about how midwives experience providing contraceptive counselling to girls and young women with ADHD. This study aimed to describe midwives’ experiences of providing contraceptive counselling to girls and young women with ADHD in Swedish youth clinics.

**Methods:**

A qualitative exploratory study design using Braun and Clarke’s reflexive thematic analysis of interviews, including ten midwives providing contraceptive counselling to young women with ADHD. The average interview time was 34 min.

**Results:**

The participating midwives were aged 31 to 62 years and had between six months and 24 years of experience working in youth clinics across four of Sweden’s six healthcare regions. Their experiences were captured by the overarching theme, *Adapting contraceptive counselling to individual needs despite limited ADHD-specific support*, which encompassed three themes: *Striving to provide person-centered counselling*, *Navigating counselling in response to increased support needs*, and *Recognizing the importance of ADHD-specific knowledge and resources to support counselling.*

**Conclusions:**

Midwives described adapting contraceptive counselling to perceived individual needs through changes in communication, consultation structures, access to care, and interprofessional collaboration. They also described a need for stronger ADHD-specific education and evidence-informed professional guidance. Support for counselling should provide a structured professional foundation while allowing flexibility to respond to individual circumstances and preferences.

**Supplementary Information:**

The online version contains supplementary material available at https://doi.org/10.1186/s12905-026-05043-4.

## Background

Attention-deficit/hyperactivity disorder (ADHD) is a neuropsychiatric condition characterised by inattention, hyperactivity, and impulsivity, according to a review published in 2020 by The Lancet [1]. In clinical practice, ADHD encompasses three presentations: predominantly inattentive (ADHD-I), predominantly hyperactive/impulsive (ADHD-HI), and combined presentation (ADHD-C) [2]. Based on a review by the Swedish National Board of Health and Welfare, the prevalence of ADHD diagnosis among girls between ten and 17 years of age is approximately 6% and continues to rise [3]. ADHD diagnosis is based on a comprehensive clinical assessment conducted within specialist healthcare according to the diagnostic criteria in the Diagnostic and Statistical Manual of Mental Disorders (DSM-5). The criteria are organized into two primary symptom domains: inattention and hyperactivity-impulsivity. The assessment includes evaluation of symptom history, functional impairment across settings, psychosocial context, developmental history, and family history [1]. For adolescents, symptoms must have been present before age 12, persisted for at least 6 months, and caused impairment in more than one setting [4].

As described by a review, ADHD is a heterogeneous condition that may present differently across individuals and across the life course [5]. Executive dysfunction, including difficulties with planning, organization, working memory, self-monitoring, and impulse control, may affect daily functioning and health-related behaviours [5]. Differential diagnoses such as trauma-related conditions, anxiety, and depression may complicate the assessment process because several symptoms overlap with ADHD manifestations [5]. Comorbid psychiatric conditions are considered one explanation for why girls and women are frequently underdiagnosed, as their symptoms may be interpreted as emotional or affective difficulties rather than ADHD itself [5]. Women and girls with ADHD often present with more inattentive symptoms, whereas boys more commonly display hyperactive and impulsive behaviours. Female presentations may therefore be less externally visible and less likely to prompt referral for assessment [5–8]. Existing diagnostic frameworks have also been criticized for being primarily based on male symptom presentations, contributing to gender bias in the diagnostic process [6–8]. In recent years, increased public awareness of ADHD through social media and online communities has also contributed to a growing phenomenon of self-identification or self-diagnosis among women who recognize previously unexplained difficulties in themselves [9]. Although self-identification may facilitate help-seeking and validation, it may also reflect persistent barriers to formal assessment and diagnostic recognition [6, 7]. Research suggests that ADHD may substantially affect girls’ and women’s quality of life, including academic performance, social relationships, emotional well-being, and self-esteem [5]. Young women with ADHD often experience low self-esteem, depression, self-harm, anxiety, and eating disorders [5, 10]. Low self-esteem may influence interpersonal relationships, risk perception, and decision-making in sexual situations, underscoring the importance of supportive and person-centered contraceptive counselling that promotes autonomy and self-efficacy [5, 9, 11].

ADHD is associated with increased engagement in unhealthy lifestyle behaviours, including substance use and sexual risk-taking behaviour. Comorbidity, sensation-seeking, and ineffective coping strategies may mediate or moderate the association between ADHD and risk-taking behaviours that increase the likelihood of adverse health outcomes [12]. Several characteristics associated with ADHD may contribute to increased sexual and reproductive health risks. Impulsivity and sensation-seeking behaviours may increase the likelihood of engaging in unplanned sexual activity, multiple sexual partnerships, or sex under the influence of alcohol or drugs [13–15]. Difficulties with executive functioning, including impaired planning, working memory, and task consistency, may reduce adherence to user-dependent contraceptive methods such as daily oral contraceptives [16, 17]. Emotional dysregulation, low self-esteem, and challenges in interpersonal relationships may further influence decision-making, boundary-setting, and vulnerability in sexual situations [5, 10, 11]. Together, these factors may increase vulnerability to adverse sexual and reproductive health outcomes, including the risk of sexually transmitted infections (STIs) [13, 14], unintended pregnancies, and sexual exploitation or abuse [5, 13, 14].

Using dual protection, such as condoms in addition to hormonal contraceptives, can reduce the risk of both pregnancy and STIs. However, condom use is decreasing, with less than half of young people reporting its use with new partners [18]. Chlamydia remains the most common STI in Sweden, particularly among women aged 20–24 years [19]. Contraceptive methods are typically classified as long-acting reversible contraception (LARC), such as intrauterine devices and implants, or short-acting reversible contraception, including pills, patches, injections, and vaginal rings, which require consistent and correct use [20]. While contraceptives are effective in preventing unintended pregnancies, their use may be influenced by perceived or experienced side effects. Hormonal contraceptives have been associated with side effects such as mood changes, irregular bleeding, and weight fluctuations, while LARC methods may involve concerns about pain during insertion or a reduced sense of bodily control [21, 22]. These factors can affect method choice, satisfaction, and continuation and may be particularly relevant for individuals with heightened sensitivity to bodily or emotional changes [21, 22].

Sexual and reproductive health and rights (SRHR) encompass physical, mental, emotional, and social well-being throughout the life course, with a focus on self-determination in matters of sexuality and reproduction [23, 24]. In practice, access to SRHR includes the ability to obtain accurate information, choose and use effective contraception, access safe abortion services, receive STI prevention and treatment, and make autonomous decisions about if and when to have children [18]. Despite Sweden’s strong public health infrastructure, targeted health promotion within SRHR requires further development to ensure equitable access, particularly for individuals with neuropsychiatric conditions [24].

Sweden’s first youth clinic opened in 1970 with the overall goal of promoting physical and mental health, with a focus on SRHR among young people. Services are based on a holistic psychosocial, psychological, and medical approach, as well as a rights-based perspective, recognizing young people’s right to information, knowledge, support, and treatment [25]. These clinics provide free services to individuals aged 12–25 years without requiring guardian involvement and address both physical and mental health, with a strong emphasis on SRHR. Interdisciplinary teams typically include midwives, physicians, psychologists, counsellors, and dietitians [26].

In Sweden, midwives play a central role in contraceptive counselling, pregnancy testing, STI testing, relationship guidance, and basic mental health support. In Sweden, midwives independently prescribe contraceptives for birth control to women and provide LARC through the insertion of contraceptive implants and intrauterine devices [25, 27]. Approximately 85–90% of all contraceptives are prescribed by midwives, and midwives are also authorized to prescribe medication for confirmed anogenital chlamydia infections [28]. To identify young individuals exposed to or at risk of sexual ill health in terms of STIs, unintended pregnancies, and sexual violence, the SEXual health Identification Tool (SEXIT) is used. The tool is composed of three components: staff training, a questionnaire for youth clinic visitors, and a written guide for staff to support subsequent dialogue and risk assessment after completion of the questionnaire [29]. Effective contraceptive counselling requires a comprehensive medical history, consideration of life circumstances, and individualized guidance to improve satisfaction and adherence [30]. Counselling should also address personal preferences, capacity for consistent use, risk factors, and the importance of STI prevention. Person-centered care—grounded in mutual respect, active listening, and shared decision-making—is essential to tailoring counselling to individual needs [31, 32].

Despite the availability of accessible contraceptive services [33], free or subsidized contraceptives are available in most of the Swedish regions [34]; however, unmet contraceptive needs persist among women aged 15–19 years in Sweden [33]. Unintended pregnancies may result from non-use, inconsistent use, or failure of contraceptive methods, as well as changing reproductive intentions [35]. These factors highlight the complexity of contraceptive decision-making and the importance of individualized, high-quality counselling. For young women with ADHD, additional challenges may arise. A systematic review found that treatment adherence among adolescents with ADHD is influenced by access to healthcare services and the quality of communication with providers. Positive provider-patient relationships and care from ADHD specialists were associated with greater adherence, whereas dissatisfaction with providers, treatment by non-specialists, and changes in healthcare providers were linked to treatment discontinuation and reduced continuity of care [36]. Reliable and trusting healthcare support is therefore important not only for treatment adherence but also for enabling young women with ADHD to feel comfortable discussing sexual and reproductive health and their ADHD diagnosis [37]. Swedish youth clinics serve individuals aged 12 years and older, encompassing a broad range of developmental stages. Consequently, the clinical, ethical, and counselling contexts may differ substantially between early adolescence and young adulthood. Given that young women with ADHD face increased sexual and reproductive health risks, alongside potential challenges with adherence and method satisfaction, it is essential to understand how healthcare providers address their specific needs. Although previous research has highlighted sexual and reproductive health vulnerabilities and barriers to healthcare among girls and women with ADHD, less is known about how contraceptive counselling is experienced and adapted by healthcare professionals in routine practice. In particular, there is limited knowledge of how midwives navigate communication, contraceptive preferences, access to care, and reproductive autonomy when counselling girls and young women with ADHD. Understanding midwives’ experiences may provide insight into how counselling is adapted within the organisational context of youth clinics. Therefore, this study aimed to describe midwives’ experiences of providing contraceptive counselling to girls and young women with ADHD in Swedish youth clinics.

## Methods

### Study design

Our study had a qualitative exploratory design using Braun and Clarke’s reflexive thematic analysis. The analytic process was carried out within the social constructionist epistemology to form a pattern of midwives’ experiences of contraceptive counselling for young women with ADHD in Sweden [38, 39]. This interview study was carried out following the methodological checklist of the *Consolidated Criteria for Reporting Qualitative Research* [40].

### Participants and recruitment procedure

Inclusion criteria for participation were to hold a current midwifery position at a youth clinic in Sweden, with experience in contraceptive consulting young women between the ages of 12 and 25 years with ADHD. Seven youth clinics in five cities (i.e., Stockholm, Uppsala, Linköping, Falun, and Gävle) were contacted between April and May 2025, with a study information letter via email or in person. In addition, a study invitation was published twice in a Facebook group with midwives who work at youth clinics in April and June 2025. The midwives who agreed to participate in the study, who were not previously known to the authors, were contacted via email for information about interview questions, arranging a time for the interview, and providing written informed consent. Finally, ten of 14 midwives were interviewed. The dropout of four midwives was due either to their not responding with a suitable time for an interview or to their unavailability for an interview during the study period.

### Data collection procedure

Semi-structured interviews were carried out between April 23rd and July 4^th,^ 2025, following a pilot-tested interview guide developed for the current study, which has not been previously published elsewhere (Table 1; Supplementary file 1). The two pilot interviews were conducted with midwifery students who had completed clinical placements at a youth clinic. Minor revisions were made to the wording of the interview questions based on the pilot interviews, without affecting their meaning or narrative structure. Data from the pilot interviews were excluded from the analysis. Data were audio-recorded individually via Zoom (*n* = 5) or via a mobile application (*n* = 5). The interviews were conducted according to the interview guide, and participants were asked to elaborate, clarify, and explain. Data were collected separately by two midwifery students trained in qualitative interviewing (one of them was the second author). The interviews lasted between 17 and 50 min, with an average time of 34 min. During the later interviews, recurring patterns relevant to the research aim were identified, and the dataset was considered sufficient for reflexive thematic analysis.

**Table 1:** Interview guide developed for the current study

• Please describe your experience in contraceptive counselling with young women with ADHD.
• Please describe what difficulties may arise during contraceptive counselling with young women with ADHD.
• What knowledge do you have about young women with neuropsychiatric variations in relation to counselling young women with ADHD?
• Please describe your experience with the knowledge and attitudes of young women with ADHD regarding contraception.

### Data analysis

The interview data were analyzed using inductive reflexive thematic analysis according to Braun and Clarke [38, 39], within a social constructionist epistemology, to identify patterns in midwives’ experiences (described by themes). The process involved six analytic steps. First, the audio files were transcribed verbatim by the second author, and all authors read and re-read the transcripts, noting initial analytic observations and insights to familiarize ourselves with the dataset. In the second step, the first author sorted significant text features into initial latent codes to describe the discovered pattern. The third step involved developing initial themes capturing patterns across the dataset in relation to how midwives experienced, adapted, and negotiated contraceptive counselling. In the fourth step, the developing themes were reviewed iteratively against the coded data and the full transcripts to assess their coherence, distinctiveness, and relevance to the research question. By the fifth step, the themes were distinguished, defined, and labelled (Table 2). In the last step, one overarching theme, including three themes and 11 sub-themes, described the midwives’ experiences (Table 3). The themes have been exemplified by a selection of rich and illustrative quotes to support the analytic interpretation [38, 39]. The analytic process has followed the 15-point checklist of criteria for a good thematic analysis process [41].

**Table 2:** Preliminary themes in the data analysis process

1. The midwife takes into account the unique needs in contraceptive counselling
2. A strong commitment to individual-based contraceptive counselling
3. A desire for in-depth knowledge about young women with ADHD for good counselling
4. Knowledge support can be beneficial for providing high-quality contraceptive counselling
5. It is challenging for young women to talk about their sexuality during contraceptive counselling
6. Adjusted contraceptive counselling based on increased sexual risk behavior
7. Takes co-morbidity into account in contraceptive counselling
8. Is part of a collaborative team to provide the best possible contraceptive counselling

**Table 3:** Themes on the different analytic levels

Adapting contraceptive counselling to individual needs despite limited ADHD-specific support		
Striving to provide person-centered counselling	Navigating counselling in response to increased support needs	Recognizing the importance of ADHD-specific knowledge and resources to support counselling
Adopting a respectful and non-judgmental approach	Strengthening sexual health through reproductive autonomy in counselling	Learning through clinical experience and self-directed education
Following a systematic counselling process	Building trust and adapting communication to support contraceptive decision-making	Drawing on experiential knowledge to adapt counselling
Taking action through collaboration while navigating complex care systems	Facilitating access to counselling in response to perceived individual needs Adapting counselling to side effects and contraceptive preferences	Seeking structured professional guidance in counselling Calling for evidence to support individualised counselling

### Reflexivity

The study was carried out at the Department of Women’s and Children’s Health at Uppsala University. The authors are female registered midwives with clinical experience in antenatal, intrapartum, and postpartum care. The second author became interested in midwives’ contraceptive counselling to young women with ADHD during her clinical placement during her midwifery education at Uppsala University, and chose this subject for her master’s thesis. She came to an understanding that counselling these women may be experienced as challenging for midwives. The other two authors for the current study were acting as a supervisor and examiner for the thesis, and have extensive experience in qualitative research and teaching midwifery students within sexual and reproductive health, including contraceptive counselling. The analytic interpretations were also discussed during seminars with midwifery students and teachers involved in the master’s programme at Uppsala University. These discussions functioned as opportunities for reflexive dialogue and analytic questioning rather than formal participant validation, contributing to the refinement of the thematic structure. The authors’ shared professional background as registered midwives may have facilitated understanding of clinical terminology, contraceptive counselling practices, and contextual nuances within youth healthcare. At the same time, these shared professional experiences may have influenced attention toward aspects of care considered clinically meaningful during theme construction and interpretation [38, 40]. The researchers also recognised that the study was initiated from an understanding that contraceptive counselling for young women with ADHD may be challenging for midwives. This pre-understanding, together with the interview question explicitly asking about difficulties that may arise during counselling, may have directed participants’ accounts towards challenging situations. During analysis, the researchers therefore reflected on whether the thematic account sufficiently represented other aspects of counselling, including experiences that were positive, rewarding, or professionally meaningful. The absence or relative prominence of such accounts was considered in relation to both the interview questions and the researchers’ pre-understandings.

### Ethics approval and consent to participate

The midwives were provided with written and oral study information, informing them that participation was anonymous and voluntary. They were free to withdraw from the study at any time without explanation, but no one chose to do so. Written and oral informed consent was obtained from all midwives. The interviews were coded with numbers, saved confidentially on password-protected storage at Uppsala University, and deleted upon publication. Therefore, this study does not include experiments on humans and/or the use of human tissue samples, but instead focuses on midwives’ work experiences. Midwives’ work experiences were considered non-sensitive data; therefore, no ethical approval was sought, as per Swedish national regulations [42]. However, the ethical principles outlined in the Declaration of Helsinki [43] and by the Swedish Research Council [44] were strictly adhered to. The research process was conducted in accordance with the General Data Protection Regulation (GDPR) [45].

## Results

The participating midwives were all females aged between 31 and 62 years (on average 47 years), and had worked at a youth clinic between six months and 24 years (on average ten years), at different youth clinics representing four of six healthcare regions in Sweden. The midwives had approximately 45 to 60 min for each contraceptive consultation. The midwives’ experiences in providing contraceptive counselling for girls and young women with ADHD are described through an overarching theme including three themes and 11 sub-themes. For more detailed information, see Table 3.

### Adapting contraceptive counselling to individual needs despite limited ADHD-specific support

Based on the inductive reflexive thematic analysis, the midwives’ experiences of contraceptive counselling for girls and young women with ADHD were captured by the overarching theme: *Adapting contraceptive counselling to individual needs despite limited ADHD-specific support.* The midwives described tailoring their counselling to meet the needs of women whose circumstances they perceived as not always being adequately accommodated within standard consultation structures. In doing so, they often relied on clinical experience and self-directed learning, as formal ADHD-specific education, evidence-based guidelines, and organizational support were perceived to be limited. These experiences were reflected in three interconnected themes (Table 3).

### Striving to provide person-centered counselling

The first theme is illustrated by three sub-themes describing how midwives adapted person-centred contraceptive counselling to perceived individual needs. Although they described following an overall structured consultation process, they adjusted communication, pacing, information, and the consultation environment according to the individual circumstances they encountered. They also used interprofessional collaboration when they perceived that additional support was required (Table 3).

### Adopting a respectful and non-judgmental approach

Midwives described building trust by introducing themselves, explaining the structure of the consultation, and using a calm and non-judgemental approach. They used open-ended questions to explore individual concerns and preferences, summarised information to establish mutual understanding, and encouraged women to express questions or uncertainty without fear of judgement. These practices were described as ways of creating a consultation in which women could participate in discussing contraceptive options according to their individual circumstances. The midwives tried to make the women feel comfortable expressing themselves without fear of judgment, as two midwives explained:

*They are generally a vulnerable group and more often face judgment in school and socially with friends; they may struggle to behave in a way considered “normal”*,* leading to social challenges.* #1

*To always treat all people with dignity*,* one should feel welcomed and not treated differently*. #5

### Following a systematic counselling process

Midwives described using a systematic consultation structure as a starting point while adapting the content and communication when they perceived individual needs. The initial consultation followed a routine for exploring reasons for seeking contraception, previous knowledge, preferences, and relevant aspects of the woman’s life. At the same time, midwives described modifying how information was presented by using written material, visual aids, models, simplified explanations, and different ways of organising information. Thus, a structured consultation was not described as a fixed procedure, but as a framework that could be adapted during the encounter. One midwife explained:

*When I read their medication list*,* I can understand it might be related to an ADHD diagnosis … it’s not often that they spontaneously tell me that they are on medication.* #9

The midwives asked each woman why they wanted contraception, if they had searched for information on contraception methods, if they had any questions about the search, and how they were generally managing in life. All these questions were aimed at finding the best method for each woman, ensuring they feel comfortable with their choice. One midwife said:

*It’s crucial to have a contraceptive method that one feels secure with and won’t forget to take*,* such as a contraceptive pill.* #2

The midwives did not have any specifically tailored informational material for women with ADHD. Instead, they adopted various strategies to inform, explain, clarify, and specify methods of contraception for everyone who came for consultation. The midwives utilized verbal and written patient information when discussing contraceptives. They used visual aids such as a birth control pill chart to show the effectiveness of different contraceptives in preventing pregnancy. They also provided images explaining the menstrual cycle, the build-up of uterine mucosa before menstrual bleeding, as well as illustrations of intrauterine devices, implants, and gynecological anatomy. To make the information more accessible, they displayed different types of contraceptives on a board for women to see, used a small uterus model to explain its location and function, and offered them to see and touch the different contraceptive devices. Additionally, to help women with attention and concentration, they used memory aids, like mind maps and jointly written lists to visualize and organize key concepts and their interrelationships. Two midwives added:

*I treat everyone in the same way*,* provide explicit information through both written and oral means*,* so that I know I’m not missing anyone with ADHD; not everyone tells me that they have it*. #5

*There should be something shorter and simpler*,* in bullet points*,* making it easier to read*. #8

One challenge associated with ADHD in contraceptive counselling was described as difficulties considering the consequences of one’s actions. The midwives perceived that this could increase vulnerability to situations involving sexual risk-taking, such as difficulties setting boundaries, being easily influenced by others, seeking attention or validation, engaging in sexual self-harming behaviors, and being at greater risk of exploitation. They also described circumstances involving non-consensual sexual experiences and unprotected sex, which may increase the risk of STIs and unintended pregnancies. Some young women are very open about their sexual self-harming behaviors, expressing indifference toward their actions, for example by stating: *“I don’t care” and “No*,* I don’t derive much pleasure from it*” (#4). Further, the midwives mentioned that they discussed sexual health and intimate violence, utilized the SEXIT; *We are here to assist them*,* and if they do not want to tell me*,* they do not have to; they might feel embarrassed* (#9). However, a midwife noted that it could be that women with ADHD are isolated rather than engaging in risky behaviors. She described how these individuals often did not have a sexual life, avoided socializing, and preferred to stay at home. This midwife said: *How can we support them in meeting and daring to talk to someone new? … It’s quite tricky* (#6).

### Taking action through collaboration while navigating complex care systems

Midwives described interprofessional collaboration as a way of extending and adapting contraceptive counselling when they perceived that needs could not be addressed within the consultation alone. They described contacting physicians, psychologists, school health nurses, social workers, social services, psychiatric services, and habilitation services depending on the circumstances. At the same time, fragmented responsibilities and multiple professionals could make continuity and communication more difficult. Collaboration therefore functioned both as a resource for individualised care and as a source of organisational complexity. Two midwives described:

*They might not want their parents to know that they are having sex*,* and then the school nurse might be helpful*. #4

*I think it should be easier to collaborate with other professionals… we have young women who share a bit here about one thing and a bit there about another thing… not getting the right help because there are so many actors involved in their care… it becomes a “hullabaloo” and then they forget who they are supposed to meet*. #3

### Navigating counselling in response to increased support needs

Midwives described adapting contraceptive counselling to women’s individual circumstances and preferences. They encountered substantial variation in women’s knowledge, contraceptive experience, sexual circumstances, and support needs. When midwives perceived concerns related to inconsistent use, sexual health, or vulnerability, they described providing additional information, adapting communication, and discussing alternative contraceptive methods. Long-acting methods were sometimes recommended when consistent use of user-dependent methods was perceived as difficult, while midwives also emphasised that women retained the right to choose another method. This created a tension between perceived contraceptive effectiveness, clinical recommendations, and reproductive autonomy (Table 3).

### Strengthening sexual health through reproductive autonomy in counselling

In counselling, the midwives sought to promote sexual health by tailoring their approach to each young woman’s capabilities, support needs, life experiences, and level of interest in sexuality and contraception. Some young women were described as requiring additional support, particularly those with comorbid mental health conditions. Two midwives explained:

*They may share bits of information with different professionals … leading to inadequate help due to disjointed care … it becomes overwhelming*. #3

*Yes*,* I believe it* [support] *may be necessary*,* … many have developed other mental health issues … anxiety*,* depression*,* eating disorders*,* or OCD* [Obsessive-Compulsive Disorder] *… therefore*,* I think they frequently require more support*. #1

Counselling was adapted to young women’s knowledge about sexuality and contraception. While some were well informed, others had limited knowledge or relied on misinformation from peers, social media influencers, or platforms, such as TikTok.

[The knowledge] *should come from school*,* but unfortunately not … they don´t understand how their menstrual cycle works and how contraceptives can affect their hormones.* #3

Midwives adapted counseling to support the young women’s decision-making, communication, and attention. They perceived that women receiving pharmacological treatment for ADHD or ongoing psychosocial support were often better able to engage in counselling. Midwives also expressed concerns about adherence to user-dependent contraceptive methods, such as oral contraceptives, citing inconsistent use and misunderstandings regarding correct use. As a result, long-acting reversible contraceptives were frequently recommended. One midwife explained:

*I forgot to take my birth control pills for three days*,* so I’ll take two now … it should be fine*,* I should still be protected*. #3

Midwives informed women about the pros and cons of various contraceptive methods and provided counselling on other available methods tailored to individual preferences. They emphasized the importance of preserving women’s autonomy in decision-making, despite sometimes experiencing tension between clinical recommendations and women’s own contraceptive preferences:

*They have the right to make decisions about their bodies. Sometimes it can be quite frustrating*. #6

Midwives described that young women with ADHD could experience difficulties maintaining attention during consultations. Some were perceived as becoming overwhelmed or losing focus after a relatively short period, which could affect their ability to engage with information and decision-making during the counselling session, as one midwife explained:

*“It feels like you’re not keeping up anymore”*,* or “It feels like you’re fading out a bit” … “How do we proceed?”* #6

Midwives adapted their counselling strategies by simplifying information, prioritizing key messages, and modifying the consultation environment to better support attention and engagement. These adaptations included minimizing distractions, using visual aids, offering breaks, and allowing movement during the consultation. One midwife described:

*I replaced my old clock with a new one that is silent.* #5

Midwives also described actively involving the women in the consultation by asking about their preferences for receiving information and offering follow-up visits when needed to support understanding and decision-making.

### Building trust and adapting communication to support contraceptive decision-making

Midwives described adapting communication to support women’s understanding and participation in contraceptive decision-making. They used questions, repetition, summaries, and opportunities for follow-up to explore preferences and concerns. At the same time, they described uncertainty about whether women were satisfied with the counselling because satisfaction was generally inferred from the interaction rather than directly assessed. One midwife explained further:

*Some are open to using hormones and the contraceptive implant… others are more skeptical… afraid of experiencing side effects… It’s a difficulty… They are already skeptical about using medications in general.* #3

Sometimes, midwives described some women as unreachable and unable to discern why the meeting did not go well. They openly invited them to return to the same or another midwife if they wished to change. Two midwives said:

*If I have been dealing a lot with someone having difficulties finding a suitable contraceptive method*,* they may have become a little irritated. Yes*,* it can lead to somewhat difficult situations; you want to help them*,* but you get nowhere*,* and it becomes frustrating*. #8

*When they come to me… “That other midwife nagged so much about this contraception*,* but I don’t want anything in my body.” Sometimes they want to switch midwives because they don’t think it went well. I explain*,* “This is your choice; I want to give you the information so you can decide for yourself.” “I won’t force you into anything. If I suggest something else*,* it’s still you who chooses.” … then they relax*. #3

Most midwives described that they were able to establish a positive connection with young women with ADHD by adapting their communication style and creating a relaxed, trusting atmosphere. They emphasized the importance of explaining information clearly, using simple language, and normalizing difficulties in understanding or remembering information. For example, one midwife described adapting her communication style:

*I have worked for a long time*,* I know what I’m talking about*,* and I can explain it in different ways. I believe they feel that I can handle the situation… and that they are receiving good and relevant information.* #1

Another midwife illustrated how self-disclosure and humor were used to build rapport and reduce pressure:

*If I notice something … I can joke about it… “I don’t always understand these things*,* so we’ll go through it step by step”… Then they usually relax…* #6

Midwives described using humor, self-disclosure, repetition, and simplified explanations to create a relaxed atmosphere and facilitate communication during consultations. Several experienced that these approaches helped young women feel more comfortable and supported during contraceptive counselling. Some midwives also described that young women occasionally contacted the youth clinic for concerns extending beyond contraceptive counselling, suggesting that the clinic was perceived as a safe and accessible point of contact. As one midwife explained:

*They would show up for anything … “My toe hurts.” … they come here first.* #3

### Facilitating access to counselling in response to perceived individual needs

Midwives described adapting access to counselling when they perceived that conventional appointment structures did not always work for individual women. They used reminders, drop-in appointments, flexible booking, telephone and digital contacts, and in some cases supported women in arranging appointments. These adaptations were described as ways of making counselling more accessible within the existing youth-clinic organisation. Three midwives added:

*It’s just the way it is. They never show up for their booked appointments.* #3

*… forgetting the appointment … getting tired of waiting … thinking about other things. But the positive side is that we have a drop-in reception every day*. #7

*Forgetting their appointments … I call them an hour before the meeting*,* to remind them*. #8

Efforts had been made to ensure easily accessible youth health care. The midwives believed it was important for the clinic to be easy to find, and they described several ways for young people to contact them. They had generous opening hours during both day and evening hours on weekdays and Saturdays, and offered scheduled, drop-in, or emergency visits. Consultations could be made in person, by phone, or digitally via chat, video, and the national healthcare link (i.e., https://www.1177.se/). Young people were sometimes described as having difficulty booking an appointment, and this was addressed by offering web-based slots or assisting them in booking an appointment on-site or via phone. The women wanted to quickly get an appointment; therefore, emergency and drop-in times were offered, but the appointment times for these meetings were short, which could be perceived as challenging. Two midwives explained:

*During drop-in visits*,* we are extra sensitive to signs* [of ADHD] … *that maybe girls will show up who have trouble keeping their appointments and easily forget*. #9

*They want to come directly when they have a question … and if they have to wait*,* they might forget about the scheduled appointment or lose interest.* #7

### Adapting counselling to side effects and contraceptive preferences

Midwives described contraceptive counselling as particularly complex when women experienced bleeding or other perceived side effects. They described discussing expected changes, exploring women’s experiences and preferences, and arranging follow-up when women considered discontinuing or changing a method. In some situations, midwives collaborated with physicians regarding method adjustments. Rather than describing discontinuation as a failure of adherence, these accounts illustrate how midwives negotiated contraceptive effectiveness, side-effect burden, continuation, and individual preferences during counselling.

The midwives explained the side effects of each contraceptive method and booked a follow-up visit to explore women’s experiences of contraceptive use. However, the midwives found it difficult to support the continued use of these methods despite their high effectiveness. While concerns about bleeding are common among contraceptive users in general, midwives perceived that young women with ADHD often exhibited a lower tolerance for such side effects. This was understood in relation to ADHD-related characteristics, including difficulties managing discomfort over time and challenges with delayed gratification, making the adjustment period associated with LARC particularly difficult.

In prescribing contraceptive methods, midwives described challenges related to emotional regulation and perceived sensitivity to hormonal changes among young women with ADHD. While mood-related side effects were considered common among contraceptive users generally, the midwives perceived them as more pronounced and more difficult to manage in women with ADHD. This contributed to skepticism toward and concerns about hormonal contraceptives, complicating both counselling and contraceptive decision-making. One midwife highlighted the difficulty of supporting continued contraceptive use when side effects occurred:

*That poses a challenge for us. “How can we motivate them to continue despite experiencing temporary side effects?”* #4

To address these concerns, midwives emphasized providing education about expected hormonal changes when initiating contraceptive methods. In some cases, symptoms resembling premenstrual dysphoric disorder were reported to worsen temporarily following the initiation of hormonal contraception. When more severe reactions occurred, midwives collaborated with physicians, who adjusted treatment, for example, by prescribing higher hormone doses, which were perceived to have a stabilizing effect.

Additional challenges arose when hormonal contraceptives were perceived to be incompatible with medications for ADHD or comorbid conditions such as depression. In these situations, midwives referred patients to physicians, as prescribing is limited to sexually active individuals without complicating medical conditions. This further highlights the complexity of contraceptive counselling for this group and the need for interprofessional collaboration.

### Recognizing the importance of ADHD-specific knowledge and resources to support counselling

The third theme describes how midwives developed and sought knowledge to support individually adapted contraceptive counselling when formal ADHD-specific education and organisational guidance were perceived as limited. Midwives described learning through clinical experience, supervision, colleagues, personal experience, courses, guidelines, and research. Midwives described a need for formal guidance to support consistent contraceptive counselling when ADHD-specific knowledge was limited (Table 3).

### Learning through clinical experience and self-directed learning

Midwives described varying levels of formal education on ADHD, with several reporting limited or no training during their nursing or midwifery education. Despite this, they frequently encountered young women with ADHD in clinical practice, requiring them to develop competence through experience and self-directed learning. Additional knowledge was acquired through seminars, supervision, interdisciplinary collaboration, and informal exchanges with colleagues. This learning included strategies for adapting communication, such as simplifying information, using visual aids, and structuring consultations to improve understanding and engagement. A midwife emphasized the importance of ongoing support:

*We meet with a supervisor once a month to discuss cases and situations… It’s good to receive knowledge support in that way*. #2.

Two midwives explain their lack of professional training regarding ADHD as follows:

*It’s not something I received through my nursing and midwifery education or in my professional life. #1*.

*No*,* I haven’t*,* but we have a physician in our region who is very dedicated to girls with ADHD … we haven’t taken any courses*,* just received some internal information at my workplace.* #4.

Another two midwives described their education on ADHD as:

*We have to undergo a neuropsychiatric disabilities certification at work*,* where we learn about autism and ADHD.* #3

*I have learned about ADHD and autism through a course. I strive to expand my knowledge beyond my midwifery education*. #5

### Drawing on experiential knowledge to adapt counselling

Some of the midwives had personal experiences with ADHD, either through themselves, their children, grandchildren, relatives, or close ones having a functional neurologic disorder. This personal connection led to a deeper understanding of the challenges and the help available. Some midwives mentioned taking courses on sexuality, mental health, intellectual disabilities, and supporting parents of children with functional neurologic disorders. A midwife said;

*I have children with neuropsychiatric disabilities*,* so I have personal experience. I have also taken a private course as a parent of children with neuropsychiatric disabilities. Thanks to my children*,* I believe I have become a better caregiver. Such courses would be really beneficial for gaining understanding because without contact with people with ADHD*,* one does not comprehend the issues. It takes quite a long time before you understand*. #1

### Seeking structured professional guidance in counselling

The midwives noted that their workplace lacked written guidelines specifically addressing contraceptive counselling for young women with ADHD. Instead, they relied on a general guideline that emphasized meeting with young people individually and adapting counselling according to their level of maturity. Several midwives expressed a need for more structured guidance, while emphasizing that such guidance should remain flexible, rather than replace, clinical judgment. As two midwives explained:

*I can have use of basic information*,* but not that one always has to follow it strictly* (#2)

*I don’t know if guidelines would have helped me. I think it’s difficult to make general statements about ADHD because it can vary so greatly.* #1

In the absence of guidelines, midwives described a tension between wanting structured professional guidance and recognizing that the considerable heterogeneity among young women with ADHD limits the feasibility of standardized counseling. They therefore sought clearer professional support rather than a uniform counseling strategy.

### Calling for evidence to support individualised counselling

Most of the midwives described research on ADHD as neglected and expressed a need for more scientific knowledge about the care in relation to the diagnosis. This includes understanding how to handle consultation situations, recognizing how hormonal fluctuations can impact ADHD symptoms and mental health, comprehending what the cognitive and executive functions need for sexual health, and determining what support may be necessary. The information they currently have focuses on the increased risk of STIs and drug addiction in relation to ADHD. A midwife added:

*More research is needed on girls with ADHD in general*,* especially regarding their sexuality. Currently*,* most research focuses on the risk associated with ADHD in girls*,* such as increased risk of STIs and drug use. There is a lack of information on how we can improve care and support for these girls. Conducting a comprehensive study on this topic would be beneficial.* #6

## Discussion

### Main findings and contribution

This study aimed to describe midwives’ experiences of contraceptive counselling for girls and young women with ADHD at Swedish youth clinics. The findings show that midwives strive to provide person-centred contraceptive counselling while navigating perceived ADHD-related complexities and limited access to ADHD-specific knowledge and resources. Rather than applying a single approach to counselling, they described adapting communication, consultation structure, follow-up practices, and contraceptive discussions to meet each young woman’s individual needs. These adaptations were made in the context of uncertainty regarding how young women experienced the counselling and how best to address ADHD-related needs. The findings therefore extend previous research by illustrating how midwives seek to translate knowledge about ADHD and sexual and reproductive health (SRH) into everyday contraceptive counselling practice.

Previous research has identified challenges in contraceptive counselling for young women with ADHD, including difficulties related to attention, communication, adherence, and contraceptive use, and has highlighted the need for improved knowledge and organisational support among midwives [46]. The present study confirms several of these challenges but also illustrates how midwives actively respond to them in clinical practice. This is consistent with more recent research showing that healthcare professionals experience difficulties in meeting the SRH needs of young women with ADHD, particularly in relation to sexuality, sexual risk-taking, relationships, and communication [47]. At the same time, the present findings add a more practice-oriented perspective by showing how midwives negotiate these challenges during contraceptive consultations rather than only describing the challenges themselves.

### Responding to needs identified by young women with ADHD

The findings should also be considered alongside research describing SRH from the perspective of young women with ADHD themselves. Young women have described challenges related to sexual relationships, sexual behaviours, self-understanding, and experiences of being different or misunderstood, as well as experiences of sexual victimisation [48]. In a later study, both young women with ADHD and healthcare professionals described reliable support as important for promoting SRH, with accessible information, trustful relationships, clear structure, early support, and appropriate organisational conditions identified as important components [37].

Several aspects of the present findings correspond with these previously described needs. The midwives emphasised the importance of a respectful and non-judgemental approach, clear structure, adapted communication, and sufficient attention to the individual young woman. Their descriptions of using structured counselling and adjusting the amount and form of information may therefore be understood as attempts to create the type of reliable and accessible support previously described by young women with ADHD and healthcare professionals [37, 48]. This correspondence is important because it suggests that adaptations described by midwives are not only responses to perceived clinical difficulties but may also address needs identified in research involving young women themselves. However, these represent different perspectives and should be interpreted accordingly. The women’s accounts describe how they experienced their own SRH and encounters with healthcare, whereas the present study captures how midwives interpreted and responded to perceived needs. Consequently, the present findings may indicate how healthcare professionals attempt to respond to needs previously described by young women with ADHD, but they cannot establish whether these adaptations were experienced by the women as supportive, accessible, or non-judgemental. Further research directly examining young women’s experiences of adapted contraceptive counselling is therefore needed [37, 48].

### From identified challenges to practical adaptations

The midwives’ descriptions of adapting the consultation are broadly consistent with previous research from Swedish youth clinics. Klint Carlander et al. [46] identified challenges related to attention, communication, treatment, and adverse effects, while also describing active adaptations in contraceptive counselling and a readiness for organisational improvement. The present findings extend this work by illustrating how such adaptations are enacted in everyday practice. Midwives described structuring consultations, adjusting the pace and amount of information, repeating or clarifying information, and using different ways of communicating depending on the individual young woman.

These findings are also consistent with Wallin et al. [37], who found that clear structure during clinical encounters could make information more accessible and that sufficient time, ADHD and SRH education, and variation in communication tools may facilitate support [37]. The convergence between these studies suggests that accessibility may depend not only on whether contraceptive information is available, but also on how information is organised and communicated during the consultation.

An important implication is that adaptation should not be understood as applying a fixed set of ADHD-specific communication techniques to all women with ADHD. ADHD is heterogeneous, and individual needs may vary according to symptom presentation, co-occurring conditions, previous healthcare experiences, sexual and relational circumstances, and the type of contraceptive counselling required. The midwives’ accounts instead indicate a process of assessing and adapting the consultation to the individual. This supports an approach in which ADHD-specific knowledge functions as a resource for understanding possible needs rather than as a basis for making assumptions about an individual woman.

### Sexual health, vulnerability and contraceptive use

The findings concerning sexuality and contraceptive use further demonstrate the complexity of contraceptive counselling in this group. Midwives described situations in which they perceived concerns related to sexual vulnerability, boundary-setting, or unprotected sex. They responded by using counselling to explore sexual health, consent, intimate violence, and contraceptive protection, and some described using SEXIT [29] as a structured way of opening such conversations.

These findings are relevant in light of previous research in which young women with ADHD have described difficulties in sexual relationships and experiences of sexual victimisation [48]. Healthcare professionals have similarly described concerns related to sexual risk-taking, complex romantic relationships, and difficulties in discussing sexuality with young women with ADHD [47]. The present findings therefore reinforce the importance of contraceptive counselling as part of broader SRH care rather than as a consultation focused solely on method selection. At the same time, counselling about sexual vulnerability requires particular attention to avoiding assumptions that ADHD inevitably results in risky sexual behaviour. The midwives’ accounts concern situations they perceived in clinical encounters and should therefore not be interpreted as evidence that young women with ADHD generally engage in unsafe or problematic sexual behaviour. Instead, the findings indicate that midwives may need to create opportunities for discussions about consent, sexual boundaries, relationships, and protection when these issues are relevant to the individual woman. This approach may help maintain a person-centred focus while ensuring that potentially sensitive aspects of SRH are not overlooked.

The findings related to forgetfulness, missed appointments, and difficulties maintaining contraceptive use also need to be interpreted carefully. Previous research has identified adherence and contraceptive management as challenges in this population [46]. However, these challenges do not necessarily imply that a particular contraceptive method is preferable for all women with ADHD. Contraceptive counselling should instead consider the individual woman’s preferences, reproductive intentions, experiences of side effects, capacity and willingness to manage a method, and need for follow-up. Long-acting reversible contraception may reduce the need for user-dependent action for some women, but should not replace individualised counselling or be presented as a universal solution [46].

### Accessibility is more than access to services

The Swedish youth-clinic context is important when interpreting these findings. Youth clinics provide relatively accessible routes to SRH counselling and contraception for adolescents and young adults, creating organisational conditions in which midwives can adapt consultations to individual needs. Previous research from Swedish youth clinics has nevertheless identified a need for greater ADHD-specific knowledge and evidence-based support among midwives [46].

Recent population-based Swedish evidence further complicates the assumption that young people with ADHD primarily experience SRH inequalities because they do not access healthcare. In a large population-based study of 454, 405 young people aged 12–22 years in Stockholm County, young people with ADHD had higher use of SRH services than peers without ADHD, including both in-person and digital contacts. The authors concluded that the relatively high level of SRH service use among young people with ADHD challenges assumptions that low healthcare engagement alone explains adverse SRH outcomes [49].

Together with the present findings, this suggests an important distinction between access to healthcare and accessibility within healthcare encounters. A young woman may successfully reach a youth clinic and receive contraceptive counselling while still encountering difficulties in processing information, communicating preferences, remembering recommendations, or obtaining follow-up that is responsive to her needs. The challenge may therefore not only be whether services are available, but whether the organisation and consultation are sufficiently accessible and responsive once the young person enters care. This distinction may also be relevant beyond the Swedish youth-clinic context. The flexibility described by the midwives may partly depend on the relatively accessible and youth-oriented organisation of Swedish youth clinics. In healthcare systems where SRH services are more fragmented or where access to midwives is less direct, similar adaptations may be more difficult to provide. The present findings should therefore not be interpreted as demonstrating that the Swedish youth-clinic model can simply be transferred to other healthcare systems, but they may illustrate how organisational accessibility can create opportunities for more responsive counselling.

### From individual adaptation to organisational responsibility

A recurring finding was the midwives’ perceived need for more ADHD-specific knowledge, education, guidelines, and evidence. This corresponds closely with previous research identifying gaps in healthcare professionals’ knowledge and calling for evidence-based interventions and organisational support [37, 46, 47]. Importantly, the findings indicate that responsibility for accessible counselling cannot rest solely with individual midwives. When midwives compensate for gaps in formal guidance by developing their own strategies, the quality and consistency of counselling may become dependent on individual knowledge, experience, and interest. This creates a potential organisational vulnerability. Structured guidance, professional education, and access to evidence-based resources could instead provide a shared foundation while allowing sufficient flexibility to adapt counselling to individual needs. Such resources should support rather than standardise the consultation. A guideline that prescribes a uniform counselling pathway may risk overlooking the heterogeneity of young women with ADHD, whereas guidance that identifies possible areas of attention—such as communication, information processing, adherence, sexuality, side effects, follow-up, and consent—could help midwives recognise relevant issues without assuming that they apply to every woman. The present findings therefore support development of ADHD-informed contraceptive counselling resources that combine structure with individualisation.

The need for organisational support is also consistent with research describing reliable SRH support as dependent not only on the healthcare professional but on organisational conditions, collaboration, and the availability of appropriate information [37]. The present study consequently points towards a multi-level understanding of accessible contraceptive counselling: adaptations are made within the consultation, supported or constrained by the organisation, and influenced by the availability of professional knowledge and evidence.

Overall, the findings indicate that midwives strive to provide person-centred contraceptive counselling while adapting to perceived individual needs related to ADHD and navigating gaps in knowledge, guidance, and organisational support. The contribution of this study lies not primarily in identifying a specific counselling technique, but in showing how accessibility is negotiated across several levels of care: through communication and consultation structure at the interpersonal level, through follow-up and collaboration at the organisational level, and through education, guidelines, and evidence at the professional level. When considered together with research from young women with ADHD and previous studies of healthcare professionals, the findings suggest that accessible SRH care requires more than the availability of contraceptive services. It also requires healthcare encounters and organisational structures that enable young women to understand information, communicate preferences, participate in contraceptive decisions, and obtain appropriate support over time [37, 46, 48].

### Strengths and limitations

This study provides an in-depth exploration of midwives’ experiences of providing contraceptive counselling to girls and young women with ADHD within the Swedish youth-clinic context. The use of reflexive thematic analysis supported an interpretive exploration [38, 39] of how midwives described adapting counselling to individual circumstances and organisational conditions. The researchers’ shared professional background as registered midwives and their experience in sexual and reproductive health may have facilitated understanding of clinical terminology and contextual aspects of contraceptive counselling. At the same time, these professional positions and the researchers’ pre-understanding that counselling young women with ADHD may be challenging may have influenced both data generation and interpretation. The interview guide was provided to participants in advance, which may have facilitated reflection and preparation. However, this may also have shaped participants’ accounts, particularly because one interview question explicitly asked about difficulties arising during counselling. The challenge-oriented wording of the interview guide may therefore have contributed to the prominence of difficulties in the dataset. During the analysis, the researchers reflected on whether the thematic account sufficiently represented other aspects of counselling, including experiences that were positive, rewarding, or professionally meaningful. The relative absence of such accounts was considered in relation to both the interview questions and the researchers’ pre-understandings.

The sample consisted of ten midwives who were recruited from youth clinics in five cities and four of Sweden’s six healthcare regions. The relatively small and self-selected sample may have resulted in participants with particular interest or experience in ADHD being more likely to participate. Differences in clinical experience, education, and organisational context may also influence how contraceptive counselling is provided. Transferability therefore depends on the similarities between the study context and other healthcare settings rather than statistical generalisation.

Finally, the study represents the perspectives of midwives only. Descriptions of women’s needs, responses, satisfaction, or engagement are therefore based on the midwives’ perceptions and cannot be interpreted as the women’s own experiences. The study cannot establish whether the adaptations described by midwives were helpful, acceptable, or effective from the women’s perspective, nor whether they influenced contraceptive continuation, attendance, or participation in decision-making. Future research should therefore include girls and young women with ADHD themselves, including consideration of differences across developmental stages.

### Implications for practice and policy

The findings suggest several areas that may warrant attention in practice, education, and organisation. Midwives’ accounts point to a need for professional support that enables adaptation of contraceptive counselling to perceived individual needs. This may include accessible information, flexible consultation structures, opportunities for follow-up, and interprofessional collaboration. Participants also described a need for stronger ADHD-specific education and evidence-informed professional guidance. Such guidance could provide a common professional foundation while allowing flexibility and clinical judgement, rather than prescribing a uniform counselling strategy for all girls and young women with ADHD. At the organisational level, flexible access to youth-clinic services and effective coordination between professionals may support the adaptations described by the midwives. At the educational level, ADHD and neurodiversity may warrant greater attention in midwifery education and continuing professional development, including communication and accessibility in SRH counselling. These implications should be understood as areas identified through the participants’ experiences rather than as interventions demonstrated to improve care outcomes. Future research should move beyond descriptions of professional experiences and examine how young women with ADHD experience these adaptations. In particular, studies could examine whether structured consultations, different information formats, follow-up strategies, and digital communication are associated with improved perceived accessibility, contraceptive knowledge, satisfaction, autonomy, and continuity of care. Intervention studies are also needed to determine whether ADHD-specific educational programmes, clinical guidelines, or organisational adaptations improve the quality and outcomes of contraceptive counselling. Given the recent evidence that young people with ADHD already make substantial use of SRH services [49], future research should also distinguish between barriers to entering care and barriers encountered within care.

## Conclusion

Midwives described striving to provide person-centered contraceptive counselling by adapting communication, consultation structures, and access to care in response to perceived individual needs among girls and young women with ADHD. They negotiated contraceptive effectiveness, side effects, individual preferences, and reproductive autonomy, and used interprofessional collaboration when they perceived that needs extended beyond the individual consultation.

In the absence of consistent formal ADHD-specific education and guidance, midwives also described developing their knowledge through clinical experience, self-directed learning, colleagues, and professional support. The findings highlight a tension between the need for structured professional guidance and the flexibility required for individualised counselling. Strengthening professional and organisational support may therefore help create conditions for individually adapted contraceptive counselling, while avoiding a uniform approach to ADHD.

## Supplementary Information

Supplementary Material 1.

## Data Availability

The datasets generated and analysed during the current study are not publicly available but are available from the corresponding author on reasonable request.

## Abbreviations

ADHD: Attention-Deficit/Hyperactivity Disorder
ADHD-C: Attention-Deficit/Hyperactivity Disorder-Combined presentation
ADHD-HI: Attention-Deficit/Hyperactivity Disorder-Hyperactive/impulsive
ADHD-I: Attention-Deficit/Hyperactivity Disorder-Inattentive
DSM: Diagnostic and Statistical Manual of Mental Disorders
LARC: Long-Acting Reversible Contraception
SEXIT: the SEXual health Identification Tool
SRHR: Sexual and Reproductive Health and Rights
STIs: Sexually Transmitted Infections

## References

[1] Posner J, Polanczyk GV, Sonuga-Barke E. Attention-deficit hyperactivity disorder. Lancet. 2020;395(10222):450–62. 10.1016/S0140-6736(19)33004-1. PMC7880081 31982036

[2] Musullulu H. Evaluating attention deficit and hyperactivity disorder (ADHD): a review of current methods and issues. Front Psychol. 2025;16:1466088. 10.3389/fpsyg.2025.1466088. PMC11891363 40066184

[3] The Swedish National Board of Health and Welfare. Continued sharp increase in ADHD diagnoses [In Swedish: Fortsatt kraftig ökning av ADHD-diagnoser. 2023. https://jannel.se/SocialstyrelsensNyaPrognosOmADHD.pdf. Accessed 9 Apr 2025.

[4] Immanuel J, Burden J. What is ADHD? Washington: American Psychiatric Association; 2025. https://www.psychiatry.org/patients-families/adhd/what-is-adhd/.

[5] Hinshaw SP, Nguyen PT, O’Grady SM, Rosenthal EA. Annual Research Review: Attention-deficit/hyperactivity disorder in girls and women: underrepresentation, longitudinal processes, and key directions. J Child Psychol Psychiatry. 2022;63(4):484–96. 10.1111/jcpp.13480. 34231220

[6] Oroian BA, Nechita P, Costandache G, Marusic R-I, Pătrașcu C, Szalontay A. Gender dynamics in ADHD: understanding ADHD in females from childhood to adulthood. Bull Integr Psychiatry. 2023;4(99):77. 10.36219/bpi.2023.4.09.

[7] Young S, Adamo N, Björk Ásgeirsdóttir B, Branney P, Michelle Beckett M, Colley W, … Woodhouse E. Females with ADHD: An expert consensus statement taking a lifespan approach providing guidance for the identification and treatment of attention-deficit/hyperactivity disorder in girls and women. BMC Psychiatry. 2020;20:404. 10.1186/s12888-020-02707-9. PMC7422602 32787804

[8] Mowlem FD, Agnew-Blais J, Taylor E, Asherson P. Do different factors influence whether girls versus boys meet ADHD diagnostic criteria? Sex differences among children with high ADHD symptoms. Psychiatry Res. 2019;272:765–73. 10.1016/j.psychres.2018.12.128. PMC6401208 30832197

[9] Yeung A, Ng E, Abi-Jaoude E. TikTok and attention-deficit/hyperactivity disorder: a cross-sectional study of social media content quality. Can J Psychiatry. 2022;67(12):899–906. 10.1177/07067437221082854. PMC9659797 35196157

[10] Antoniou E, Rigas NR, Orovou E, Papatrechas A, Sarella AS. ADHD Symptoms in Females of Childhood, Adolescent, Reproductive and Menopause Period. Mater Sociomed. 2021;33(2):114–8. 10.5455/msm.2021.33.114-118. PMC8385721 34483739

[11] Dehlendorf C, Krajewski C, Borrero S. Contraceptive counseling: best practices to ensure quality communication and enable effective contraceptive use. Clin Obstet Gynecol. 2014;57(4):659–73. 10.1097/GRF.0000000000000059. PMC4216627 25264697

[12] Pollak Y, Shoham R, Scheres APJ, Dekkers TJ. ADHD and risk-taking behavior: associations, mechanisms, and interventions. Chapter 12 in Clinical handbook of ADHD assessment and treatment across the lifespan, Ed. Matson JL., Springer Nature Switzerland AG. 2023; pp. 259–332. ISBN 978-3-031-41709-2 (eBook). 10.1007/978-3-031-41709-2_12

[13] Young S, Klassen LJ, Reitmeier SD, Matheson JD, Gudjonsson GH. Let’s talk about sex… and ADHD: Findings from an anonymous online survey. Int J Environ Res Public Health. 2023;20(3):2037. 10.3390/ijerph20032037. PMC9915044 36767401

[14] Skoglund C, Kallner HK, Skalkidou A, Wikström A-K, Lundin C, Hesselman S, Wikman A, Poromaa IS. Association of attention-deficit/hyperactivity disorder with teenage birth among women and girls in Sweden. JAMA Netw Open. 2019;2(10):e1912463. 10.1001/jamanetworkopen.2019.12463. PMC6777395 31577361

[15] Pettersson C. Will you judge me if I tell you? … On young people’s sexual behavior and exposure to violence [In Swedish: Kommer du att döma mig om jag berättar? om ungdomars sexuella beteende och utsatthet för våld]. [Doctoral dissertation]. Linnaeus Univ. 2023. 10.15626/LUD.449.2023. Accessed 18 Aug 2025.

[16] Barkley RA. Attention-deficit hyperactivity disorder: a handbook for diagnosis and treatment. 4th ed. The Guilford Press; 2015.

[17] Safren SA, Duran P, Yovel I, Perlman CA, Sprich S. Medication Adherence in Psychopharmacologically Treated Adults With ADHD. J Atten Disord. 2007;10(3):257–60. 10.1177/1087054706292165. 17242421

[18] The Swedish Association for Sexuality Education. Change in attitudes towards condom use in Sweden- fewer young people protect themselves [In Swedish: Trendbrott i kondomanvändningen i Sverige – färre unga skyddar sig]. 2024. https://www.rfsu.se/om-rfsu/press/pressmeddelanden/2025/trendbrott-i-kondomanvandningen-i-sverige/. Accessed 15 Apr 2025.

[19] The Public Health Agency of Sweden. Chlamydia infection – disease statistics [In Swedish: Klamydiainfektion - sjukdomsstatistik]. 2024. https://www.folkhalsomyndigheten.se/folkhalsorapportering-statistik/statistik-a-o/sjukdomsstatistik/klamydiainfektion/. Accessed 16 April 2025.

[20] Region Stockholm. Anticonception [In Swedish: Antikonception]. 2024. https://kunskapsstodforvardgivare.se/omraden/kvinnosjukdomar-och-forlossning/riktlinjer-for-bmm/barnmorskemottagning/antikonception/riktlinjer/antikonception. Accessed 21 May 2025.

[21] Skovlund CW, Mørch LS, Kessing LV, Lidegaard Ø. Association of hormonal contraception with depression. JAMA psychiatry. 2016;73(11):1154–62. 10.1001/jamapsychiatry.2016.2387. 27680324

[22] Bitzer J. Effects of hormonal contraception on mood and sexuality. Best Pract Res Clin Obstet Gynecol. 2024;97:102560. 10.1016/j.bpobgyn.2024.102560. 39424518

[23] The Swedish National Board of Health and Welfare. Sexual and reproductive health and rights [In Swedish: Sexuell och reproduktiv hälsa och rättigheter (SRHR)]. 2014. https://www.socialstyrelsen.se/kunskapsstod-och-regler/omraden/sexuell-och-reproduktiv-halsa-och-rattigheter/. Accessed 14 April 2025.

[24] The Public Health Agency of Sweden. Sexual and reproductive health and rights (SRHR) [In Swedish: Sexuell och reproduktiv hälsa och rättigheter (SRHR)]. 2014. https://www.folkhalsomyndigheten.se/livsvillkor-levnadsvanor/sexuell-halsa-hivprevention/sexuell-och-reproduktiv-halsa-och-rattigheter/. Accessed 1 Apr 2025.

[25] The Association for Sweden’s Youth Clinics. Handbook for Sweden’s youth clinics [In Swedish: Handbok för Sveriges ungdomsmottagningar]. 202.4 https://fsum.nu/wp-content/uploads/2024/05/handbok-24.pdf. Accessed 18 Aug 2025.

[26] Sandler Å. About the youth clinic. 2023. https://www.umo.se/att-ta-hjalp/ungdomsmottagningen/om-ungdomsmottagningen/. Accessed 14 Apr 2025.

[27] Thomée S, Malm D, Christansson M, Hurtig A-K, Wiklund M, Waenerlund A-K, Goiolea I. Challenges and strategies for sustaining youth-friendly health services—a qualitative study from the perspective of professionals at youth clinics in northern Sweden. Reprod Health. 2016;13(1):147. 10.1186/s12978-016-0261-6. PMC5178097 28003025

[28] The Swedish Association of Health Professionals. Contraceptive prescribing rights for midwives [In Swedish: Förskrivningsrätt för barnmorskor]. https://www.vardforbundet.se/rad-och-stod/regelverket-i-varden/forskrivningsratt/forskrivningsratt-for-barnmorskor/. Accessed 20 September 2026.

[29] Hammarström S. Identification of young people at risk of sexual ill health: Implementing a new tool in youth clinics. Volume 1784. Linköping University; 2021. 10.3384/diss.diva-178042. Doctoral dissertation. Accessed 28 June 2025.

[30] The Swedish Medical Product Agency. Contraception treatment recommendations [In Swedish: Antikonception- behandlingsrekommendation]. 2014. https://www.lakemedelsverket.se/globalassets/dokument/behandling-och-forskrivning/behandlingsrekommendationer/behandlingsrekommendation/behandlingsrekommendation-antikonception.pdf. Accessed 1 Apr 2025.

[31] Hedberg B, Wijk H, Andersson Gäre B, Petersson C. Shared decision-making and person-centred care in Sweden: Exploring coproduction of health and social care services. Z Evid Fortbild Qual Gesundhwes. 2022;171:129–34. 10.1016/j.zefq.2022.04.016. 35610136

[32] Ekman I, Lundberg M, Lood Q, Swedberg K, Norberg A. Person-centering – An ethics in practice. In: Ekman I, editor. Person-centering in health and medical care: From philosophy to practice. [In Swedish: Personcentrering inom hälso- och sjukvård Från filosofi till praktik]. Stockholm: Liber AB; 2020. pp. 27–53.

[33] The Swedish National Board of Health and Welfare. Statistics on abortions [In Swedish: Statistik om aborter]. 2023. https://www.socialstyrelsen.se/statistik-och-data/statistik/alla-statistikamnen/aborter/. Accessed 15 April 2025.

[34] The Swedish Organization for Obstetricians and Gynecologists. Sexual and Reproductive Health in Adolescents [In Swedish: Sexuell och reproduktiv hälsa hos ungdomar]. https://www.sfog.se/wp-content/uploads/2025/05/arg_69a4c0eb71-248d-4073-acea-f749b088dbac.pdf. Accessed 15 Ap 2025.

[35] Bitzer J, Marin V, Lira J. Contraceptive counselling and care: a personalized interactive approach. Eur J Contracept Reprod Health Care. 2017;22(6):418–23. 10.1080/13625187.2017.1414793. https://www.tandfonline.com/doi/abs/. 29322814

[36] Karimi M, Ghasemzadeh S, Fami FS. Treatment Adherence in ADHD: A Systematic Review of Influencing Factors and Strategies for Improvement. J Atten Disord. 2026;30(5):590–614. 10.1177/10870547251387006. 41267417

[37] Wallin K, Wallin-Lundell I, Alehagen S, Hanberger L, Hultsjö A. Having reliable support: A prerequisite to promote sexual and reproductive health in young women with ADHD. Arch Sex Behav. 2024;53:4117–29. 10.1007/s10508-024-03001-5. PMC11588869 39313694

[38] Braun V, Clarke V. Thematic Analysis: a practical guide. Newbury Park, CA: Sage; 2022.

[39] Braun V, Clarke V. What can thematic analysis offer health and wellbeing researchers? Int J Qual Stud Health Well-being. 2014;9:26152. 10.3402/qhw.v9.26152. PMC4201665 25326092

[40] Tong A, Sainsbury P, Craig J. Consolidated criteria for reporting qualitative research (COREQ): a 32-item checklist for interviews and focus groups. Int J Qual Health Care. 2007;19(6):349–57. 10.1093/intqhc/mzm042. 17872937

[41] Braun V, Clarke V. Successful qualitative research: a practical guide for beginners. London: SAGE Publications Ltd; 2013.

[42] The Swedish Ethical Review Authority regulation. Protecting the individual in research. https://etikprovningsmyndigheten.se/faq/vad-innebar-det-sa-kallade-studentundantaget/. Accessed 29 Nov 2025.

[43] World Medical Association. 2024. WMA Declaration of Helsinki- Ethical principles for medical research involving human participants. https://www.wma.net/policies-post/wma-declaration-of-helsinki/. Accessed 26 Nov 2025. 10.1001/jama.2024.21972 39425955

[44] Swedish Research Council. Good Research Practice 2024. Report 2025. Stockholm: Sweden. https://www.vr.se/english/analysis/reports/our-reports/2025-07-03-good-research-practice-2024.html. Accessed 26 November 2025.

[45] Wolford B. What is GDPR, the EU’s new data protection law? GDPR.EU. 2018. https://gdpr.eu/what-is-gdpr/#:~:text=The%20General%20Data%20Protection%20Regulation%20%28GDPR%29%20is%20the,collect%20data%20related%20to%20people%20in%20the%20EU. Accessed 26 Nov 2025.

[46] Klint Carlander A-K, Thorsell M, Demetry Y, Nikodell S, Kopp Kallner H, Skoglund C. Knowledge, challenges, and standard of care of young women with ADHD at Swedish youth clinics. Sex Reprod Healthc. 2022;32:100727. 10.1016/j.srhc.2022.100727. 35461165

[47] Wallin K, Alehagen S, Hanberger L, Wallin Lundell I, Hultsjö S. Sexual and reproductive health in young women with ADHD from the view of health care professionals. BMC Womens Health. 2024;124389. 10.1186/s12905-024-03230-9. PMC11225155 38970031

[48] Wallin K, Wallin Lundell I, Hanberger L, Alehagen S, Hultsjö S. Self- experienced sexual and reproductive health in young women with Attention Deficit Hyperactivity Disorder: a qualitative interview study. BMC Womens Health. 2022;22(1):289. 10.1186/s12905-022-01867-y. PMC9281117 35836208

[49] Hellsten L, Rast J, Nielsen A, Ekström AM, Gemzell-Danielsson K, Kosidou K, Ahlqvist VH. Sexual and Reproductive Healthcare Among Youth With Autism and ADHD: A Population-Based Study From Sweden. Autism Res. 2026;e70306. 10.1002/aur.70306. PMC13599709 42522081

[50] Swedish Riksdag. The Swedish Act on Ethical Review of Research Involving Humans SFS. 2003:460). https://rkrattsbaser.gov.se/sfst?bet=2003:460. Accessed 29 Nov 2025.

