# Supplementary material for: “One counselling strategy does not fit all”: Midwives’ experiences of providing contraceptive counselling to girls and young women with ADHD - a Swedish qualitative interview study
Source: BMC Womens Health. 2026 Oct 26;26:570. doi: 10.1186/s12905-026-05043-4 (PMC13718115; doi:10.1186/s12905-026-05043-4)
Supplement: Supplementary file 1 [file 12905_2026_5043_MOESM1_ESM.docx]

**Box 1.** Interview guide developed for the current study

- Please describe your experience in contraceptive counseling with young women with ADHD.
- Please describe what difficulties may arise during contraceptive counseling with young women with ADHD.
- What knowledge do you have about young women with neuropsychiatric variations in relation to counselling young women with ADHD?
- Please describe your experience with the knowledge and attitudes of young women with ADHD regarding contraception.
